# Improving the Properties of Degraded Soils from Industrial Areas by Using Livestock Waste with Calcium Peroxide as a Green Oxidizer

**DOI:** 10.3390/ma14113132

**Published:** 2021-06-07

**Authors:** Angelika Więckol-Ryk, Maciej Thomas, Barbara Białecka

**Affiliations:** 1 Department of Risk Assessment and Industrial Safety, Central Mining Institute, Plac Gwarków 1, 40-166 Katowice, Poland; 2 Chemiqua Water & Wastewater Company, Skawińska 25/1, 31-066 Kraków, Poland; 3 Department of Environmental Monitoring, Central Mining Institute, Plac Gwarków 1, 40-166 Katowice, Poland; bbialecka@gig.eu

**Keywords:** calcium peroxide, *Escherichia coli*, poultry manure, organic fertilizer, degraded soil, response surface methodology

## Abstract

Over the past years, the treatment and use of livestock waste has posed a significant problem in environmental engineering. This paper outlines a new approach to application of calcium peroxide (CaO_2_) as a green oxidizer and microbiocidal agent in the treatment of poultry manure. It also presents the application of pretreated waste in improvement of degraded soils in industrial areas. The CCD (Central Composite Design) and RSM (Response Surface Methodology) were employed for optimizing the process parameters (CaO_2_ concentration 1.6–8.4 wt %, temperature 5.2–38.8 °C and contact time 7–209 h). The analysis of variance (ANOVA) was used to analyze the experimental results, which indicated good fit of the approximated to the experimental data (R^2^ = 0.8901, R^2^*_adj_* = 0.8168). The amendment of CaO_2_ in optimal conditions (8 wt % of CaO_2,_ temperature 22 °C and contact time 108 h) caused a decrease in bacteria *Escherichia coli* (*E. coli*) in poultry manure from 8.7 log_10_ CFU/g to the acceptable level of 3 log_10_ CFU/g. The application of pretreated livestock waste on degraded soils and the studies on germination and growth of grass seed mixture (*Lollum perenne*—Naki, *Lollum perenne*—Grilla, *Poa pratensis*—Oxford, *Festuca rubbra*—Relevant, *Festuca rubbra*—Adio and *Festuca trachypylla*—Fornito) showed that a dose of 0.08 g of CaO_2_ per 1 gram of poultry manure induced higher yield of grass plants. The calculated indicators for growth of roots (GFR) and shoots (GFS) in soils treated with poultry manure were 10–20% lower compared to soils with amended CaO_2_. The evidence from this study suggests that CaO_2_ could be used as an environmentally friendly oxidizer and microbiocidal agent for livestock waste.

## 1. Introduction

Land degradation caused by industrial activity represents a serious environmental problem that affects the formation of degraded areas. Rehabilitation of these lands using organic waste products [1,2,3] enriches the soils with organic carbon, improving soil structure and its physiochemical properties.

The application of poultry manure (PM) as a useful soil amendment is a very common practice in agriculture and land rehabilitation [4,5]. The high content of organic matter [6], valuable nutrients such as nitrogen (N), phosphorus (P), potassium (K) and other essential elements make this waste suitable for improving the properties of affected soils [7]. Poultry waste contains both organic and inorganic forms of nutrients in their not available or bioavailable forms, the latter being suitable for plant growth and development. The composition of raw PM varies with the bird species, their age, diet (feed, ruminants and animal drugs) and the management of the waste product [8].

One of the negative aspects in the use of PM for fertilizing process is the high concentration of pathogens including bacteria (i.e., *Salmonella, Campylobacter, Yersinia*, *Listeria monocytogenes* and *E. coli*), fungi (i.e., *Aspergillus, Penicillium notatum, Penicillium* sp., *Cladosporium* sp., *Alternaria* sp. and *Candida albicans*) [9,10,11,12], viruses causing avian influenza in birds and humans (HPAI and H5N1) [4] and live eggs of intestinal parasites (*Ascaris* sp. *Trichuris* sp. and *Toxocara* sp.) [13]. The number of bacteria in fresh poultry manure may exceed 10 log_10_CFU/g and the number of fungi and molds may exceed even 9 log_10_CFU/g [14].

Moreover, PM contains heavy metals including arsenic (As), cobalt (Co), copper (Cu), iron (Fe), manganese (Mn), selenium (Se), nickel (Ni) or zinc (Zn), which are present in feeds and nutritional supplements [15]. Other disadvantageous components of PM include antibiotics the most common being tetracycline, penicillin and sulphonamides [16]. According to the Supreme Audit Office in Poland, in 2015 and 2016, using veterinary antibiotics to reduce infections of poultry was practiced by 82% of Polish poultry producers.

For the above reasons, applying large amounts of PM for agriculture may lead to accumulations of those pollutants in soils and ground waters and pose serious health risks to humans, animals and plants. One of the most widely studied pathogens in PM is *Escherichia coli* (*E. coli*) [11,17,18]. For safe use of organic fertilizers as a soil amendment, the maximum permissible threshold of *E. coli* (Enterobacteriaceae family) is 3 log_10_ CFU per gram of tested material [19].

The most common animal manure management method [20] allows one to reduce the total number of microorganisms to 3 log_10_ CFU/g is composting [21,22,23]. However, reducing pathogens by storage at temperature below 5 °C requires from six months up to one year. This relatively time-consuming process poses problems with storing hazardous livestock waste.

Another method for eliminating the pathogens from livestock wastes is the hygienizing process using the chemical compounds: calcium oxide (CaO) or calcium dihydroxide (Ca(OH)_2_) [24]. The microbiocidal effect of the method results from an increase in pH value up to 12 and temperature 50–70 °C, during the contact of calcium compounds with moisture and organic material. In such conditions, the inactivation of microorganism cells is observed [25].

The hygienization using calcium compounds is not neutral for the natural environment. This method is successful in acidic areas of low pH value, however in neutral conditions, it will make the pH value of the soil alkaline, causing the loss of nitrogen or poor bioavailability of phosphorus.

An alternative to the commonly applied calcium compounds is solid calcium peroxide with the chemical formula CaO_2_. In certain conditions this inorganic peroxide offers a source of oxygen and hydroxyl radicals.

Due to low water solubility of CaO_2_ and its reaction product Ca(OH)_2_, the oxygen generating process is very slow and allows one to release oxygen over prolonged periods [26,27]. Additionally, Ca(OH)_2_ formation during contact with moisture increases the pH value in two steps process according to the following reactions (1)–(2):

2CaO_2_ + 2H_2_O → 2Ca(OH)_2_ ↓ + O_2_ ↑ (1)

CaO_2_ + 2H_2_O → Ca(OH)_2_ ↓+ H_2_O_2_ (2)

H_2_O_2_ formed during the reaction (2) decomposes rapidly into water and oxygen according to the reaction (3):

2H_2_O_2_ → 2H_2_O + O_2_ ↑ (3)

A major advantage of CaO_2_ is that it is safe for the environment, non-toxic and easily degradable. It is a good indicator of the activity of soil or activated sludge, and as such it has been widely used as amendment to supply external oxygen in agriculture and soil bioremediation [28,29,30,31].

Several studies reported CaO_2_ to have potential for bioremediation of soils and acceleration of the removal of organic pollutions (petroleum hydrocarbons, polycyclic aromatic hydrocarbons, tetrachloroethylene, endocrine disrupting compounds, polychlorinated biphenyls, fluoranthene, 2,4,6-trinitrotoluene and others) [25,32,33,34,35,36].

The effect of use CaO_2_ on the reduction of toxicity of arsenic in soil pore water and rice plants growth with different CaO_2_ treatments was studied by Syu et al. [37].

Another advantage of using CaO_2_ is its possible contribution to elimination of tetracycline and other veterinary drugs (e.g., levamisole and albendazole) from PM before their decomposition into the soil [38,39].

The number of publications on the use of peroxides in livestock waste treatment is still limited and this problem has not been sufficiently considered. In this research, optimizing the process of hygienizing poultry manure with CaO_2_ as a microbiocidal agent and green oxidizer was applied. Several concentrations of CaO_2_ selected by using CCD/RSM were examined on laboratory scale. The impact of the dose of CaO_2_ on the number of *E. coli* in tested waste material and plant growth with two tested soils originated from degraded areas was investigated.

## 2. Materials and Methods

### 2.1. Chemicals

Calcium peroxide (technical grade, 78.1 wt % CaO_2_, Ixper^®^ 75C, Solvay Chemicals International S.A., Brussels, Belgium) was used as the microbiocidal agent. Double distilled water (< 2 µS/cm) was used in all experiments.

### 2.2. Sample Collection and Preparation

**Figure 1:**
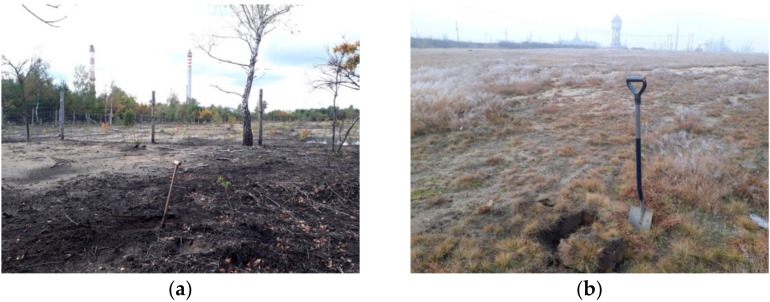
Soil sampling areas near the former “Miasteczko Śląskie” zinc smelter (**a**) and non-ferrous metals steelworks “Szopienice” (**b**).

Average analytical samples of two soils taken at a 0–15 cm depth from industrial degraded areas located in Upper Silesia in Poland were used in these investigations. One of the soils originated from Miasteczko Śląskie (S_1_) located in the nearest vicinity of a zinc smelter (Figure 1a) and the other from Szopienice (S_2_), the former area of non-ferrous metals steelworks (Figure 1b). The soil samples were air dried, crushed and passed through a 2 mm sieve. The soil samples were analyzed as described in the analytical procedures section. Four one-kilogram samples were separated from each soil. One sample of each soil without any additives was set as a control sample. The other samples were mixed thoroughly with poultry manure (PM) and a proper amount of CaO_2_. Poultry manure was collected from commercial poultry houses situated in Silesia region in Poland. The sample of PM was analyzed as described in the analytical procedures section.

### 2.3. Physicochemical Analytical Procedures

The moisture content was determined by drying samples to constant weight at 105 ± 1 °C (SLN 15, Pol-Eko-Aparatura Sp. J., Wodzisław Śląski, Poland). Ash content was determined by burning the sample at 815 °C (NABERTHERM high temperature chamber furnace HT 16/16 with a P310 controller, Nabertherm GmbH, Lilienthal, Germany). Total organic carbon (TOC) and total sulphur (S) was determined with infrared spectroscopy (ELTRA CHS, Eltra GmbH, Haan, Germany), whereas the content of nitrogen according to the conventional Kjeldahl method. The chemical composition of poultry manure sample (Al, Ca, Fe, K, Mg, Na, P, S, Si and Ti) and trace elements (Ba, Cd, Co, Cr, Cu, Mn, Ni, Pb, Rb, Sr and Zn) was determined with ICP-OES, (Perkin Elmer Optima 5300, Perkin Elmer Inc., Waltham, MA, USA) after a prior mineralization the samples in aqua regia. The content of macronutrients and trace elements in both soils were determined with the wavelength-dispersive X-ray fluorescence spectrometry method (WDXRF) after burning the samples at 815 °C (Rigaku ZSX Primus, Rigaku Analytical Devices Inc., Wilmington, NC, USA). The obtained results were reported in Table 1.

### 2.4. Microbiological Analysis

For the enumeration of *E. coli* Endo medium was used (BTL Ltd., Łódź, Poland). The decimal solutions (from 10^−1^ to 10^−7^) were made by using the Ringer’s solution and a vortex shaker was used to mix the solutions (Vortex Classic, Velp Scientifica, Usmate Velate MB, Italy). The *E. coli* enumeration was performed by adding 0.1 mL of the decimal solution sample to the Petri dishes with Endo medium. After that, the Petri dishes were incubated at 37 ± 1 °C for 24 ± 2 h. After incubation time, the circular, black and metallic shine colonies of *E. coli* were counted. Each experiment was performed in three repetitions. The mean value of the obtained results was expressed as log_10_CFU in 1 gram of the sample and calculated according to Equation (4).

(4) $$\mathrm{EC}=\frac{a}{b}\cdot {\left(10^{-x}\right)}^{-1}$$

where: a is the number of colonies of *E. coli*, b is the volume of plated sample and x is the dilution coefficient.

The analysis was conducted in the same way for treated (CaO_2_) and untreated samples.

### 2.5. Reponse Surface Methodology (RSM)

The CCD/RSM [40] was used for optimization of the microbial inactivation of poultry manure by using CaO_2_ as the microbiocidal agent. The Statistica 10 (Tibco Software Inc., Palo Alto, CA, USA) was employed to identify the most optimal conditions for lowering the concentration of *E. coli* bacteria in tested livestock waste. The plan comprised 16 experiments for three independent variables, i.e., concentration of CaO_2_ (3.0–8.4 wt %, denoted as x_1_) process temperature (5.2–38.8 °C denoted as x_2_) and contact time (7.0–209.0 h denoted as x_3_). The number of *E. coli* (denoted as Y and calculated as log_10_CFU/g) was the dependent parameter. The experiments no. 1–14 concerned changes in the value of the incoming variables in the vertices of the area, and experiments no. 15–16 concerned the middle of the 3D area, i.e., the centre of the surface and were to determine the experimental error. The verification of the significance of the given coefficients of the approximating function was conducted with ANOVA. The coefficient of determination R^2^, adjusted coefficient of determination R_adj._^2^ and the root mean square error (RMSE) of so-called fitting error variance were determined. The drawn response surface plots enabled forecasts of the changes in the estimated values, depending on the changes in the independent values. The quadratic model (5) based on the second-order polynomial equation was applied to describe the dependence between response-number of *E. coli* bacteria (Y) and the independent factors (x_1_, x_2_, x_3_):

(5) $$Y=\beta_{0}+\beta_{1}x_{1}+\beta_{1}x_{1}^{2}+\beta_{3}x_{2}+\beta_{4}x_{2}^{2}+\beta_{5}x_{3}+\beta_{6}x_{3}^{2}+\beta_{7}x_{1}x_{2}+\beta_{8}x_{1}x_{3}+\beta_{9}x_{2}x_{3}+\varepsilon$$

where: β is coefficients of the model (contribution of the independent variable in forecasts of variable Y); ε is random experimental error of normal distribution; x_1_ is concentration of CaO_2_ (wt %); x_2_ is temperature (°C); x_3_ is contact time (h).

The analysis of variance (ANOVA) was used to determine the model significance and the regression coefficient. To evaluate the fit of the model, the coefficient (R^2^) was determined, and Fisher’s F-test served to assess the statistical relevance, while contour structures of the model-expected responses and the response surface and were applied to evaluate the mutual corrections between the relevant parameters. Table 2 reports the set-up of 16 experiments obtained by using CCD. 

For the most favorable values of the three input parameters further experiments with two soils (S_1_ and S_2_) were performed.

### 2.6. Phytotest with Grass Seed Mixture

The purpose of the study was to determine the effect of the amendment of CaO_2_ -treated PM on the growth of plants in degraded soils from industrial areas.

The amount of PM (1 wt % and 2 wt %) was calculated for the treatment 5 and 10 t/ha of organic fertilizers in soils respectively. The amount of 8 wt % CaO_2_ (B) of fresh PM was applied after optimizing the parameters of inactivation process as a sufficient dose of microbiocidal agent in ambient temperature 22 °C The control soils (for S_1_ and S_2_ sample) and the following mixtures of soils and livestock waste, before and after treatment with microbiocidal agent were used in this research: soils with poultry manure (PM) in ratio 100:1 (S_1_ + 1% PM and S_2_ + 1% PM), soils with poultry manure in ratio 200:1 (S_1_ + 2% PM and S_2_ + 2% PM); soils with poultry manure in ratio 100:1 and CaO_2_ (S_1_ + 1% PM + B and S_2_ + 1% PM + B); soils with poultry manure in ratio 200:1 and CaO_2_ (S_1_ + 2% PM + B and S_2_ + 2% PM + B).

The experiments were carried out in laboratory conditions under constant temperature (22 ± 1 °C) for the entire day, controlled humidity (35% ± 5%) and lighting parameters (70 W, 4900 lm, 6000 K) with three repetitions.

Plastic plant pots with drainage holes containing 0.500 ± 0.010 kg of soil were used in this experiment. The height of the pots was 9 cm and diameter was 10 cm at the top and 7 cm at the bottom.

Each pot was watered once a day (20 mL/day) with distilled water and exposed to white light for 12 h a day. After 21 days the sprouted plants were carefully harvested, washed under running water to remove soil particles, then weighed and evaluated for the following growth parameters: length of root and shoot (cm). Then the sprouted plants were oven dried to the constant weight at 70 ± 1 °C according to the literature review [41,42,43] and weighted again. The following seeds of universal grass mixture were used in the plant test: *Lollum perenne* Naki—50%, *Lollum perenne* Grilla—15%, *Poa pratensis* Oxford—5%; *Festuca rubbra* Relevant—5%; *Festuca rubbra* Adio—5%; *Festuca trachypylla* Fornito—5% (Rolimpex SA, Iława, Poland). In each pot 0.5 g of seeds of grass mixture was placed at the depth of 1 cm.

The analysis was carried out by measuring the increase in the biomass and the length of the roots and shoots. The growth indicator of roots (GFR) was calculated according to Equation (6):

(6) $$\mathrm{GFR}=\frac{R_{S}-R_{C}}{R_{S}}\cdot 100\%$$

where: R_S_ is the average length of roots on the tested soil and R_C_ is the average length of roots on the control soil.

The growth indicator of shoots (GFS) was calculated according to Equation (7):

(7) $$\mathrm{GFS}=\frac{S_{S}-S_{C}}{S_{S}}\cdot 100\%$$

where: S_S_ is the average length of shoots on the tested soil and S_C_ is the average length of shoots on the control soil.

## 3. Results and Discussion

### 3.1. Physicochemical and Microbiological Characteristic of Soils and Poultry Manure

Table 1 presents the determined physicochemical and microbiological parameters of soil samples (S_1_, S_2_) and poultry manure (PM) used in the investigations.

The performed analysis shows that the appointed value of TOC amounting to 418.5 g/kg for PM indicates high intake of organic matter as opposed to S_1_ and S_2_ where the ash content amounted to 95.4% and 97.5% respectively. According to the literature data, silicone (Si) is one of the main elements in most soils with content ranges from 1 to 45 wt % [44]. The obtained results indicated that the content of Si for S_1_ and S_2_ were 413.8 g/kg and 441.8 g/kg, respectively. It is clear, that macro- and micronutrients included in soil are essential for the good development and quality of the plants [45]. The main macronutrients (N, P and K) for PM amounted to 56.7, 20.1 and 23.6 g/kg^-1^, respectively whereas Ca, Mg and S were 24.0, 8.5 and 6.2 g/kg.

On the one hand, the concentration of macronutrients that have a positive effect on plant growth in both soil samples was very low (0.3–7.2 g/kg for S_1_ and 0.1–4.8 g/kg for S_2_) and varied in the order of K > Ca > Na > Mg > S > P for S_1_ and K > Ca > S > P > Mg > Na for S_2_. On the other hand, soil samples contained many toxic elements that may have had an adverse environmental impact. The highest concentrations of metals were observed for Zn and Pb (6920.1 and 942 mg/kg for S_1_ and 746.3 and 387.2 for S_2_, respectively). Both soil samples S_1_ and S_2_ contained also Cd (35.2 and 12.1 mg/kg), Cr (12.1 and 13.1 mg/kg), Co (3.0 and 5.0 mg/kg) and Al (15.5 and 9.5 mg/kg).

Low concentrations of Ni, Rb and Sr were observed also for PM and soils. Macronutrients play an important role in plant metabolism by enhancing the growth and yields and protecting plants from stresses and disease [46]. Both the deficiency and the excess of macronutrients may reduce the plant growth. The most frequently observed symptoms of deficiency macronutrients are stunted growth, poorly developed root systems, reduction in leaf size, chlorosis, discoloration or necrosis. On the other hand, an excess of macronutrients may appear in the form of abnormal growth, chlorosis, leaf discoloration and necrotic spotting [47].

In fact, the micronutrients such as Fe, Mn, Cu, Zn and Mo in trace amounts are also required for proper development of plants. Some studies have shown that the content of heavy metals in raw animal manure did not affect the toxicity of the plant growth [48].

The presence of heavy metals in soil such as Pb, Cr, As, Zn, Cd, Cu, Hg and Ni may pose risks and hazards to humans and the ecosystem [49]. According to the Polish regulations [19] the threshold values of some heavy metals (Pb, Cr, Ni, Cd and Hg) in organic fertilizers (i.e., sewage sludge or animal manure) must not exceed 140, 100, 60, 5 and 2 mg/kg dry matter, respectively. The obtained results indicate that the concentration of heavy metals determined in PM was not exceeded (see Table 1).

### 3.2. CCD/RSM Results

The results of the 16 experiments performed for the combination of different values of CaO_2_ concentration, temperature and contact time are presented in Table 2. The analysis of the data showed that the lowest number of bacteria *E. coli* (3 log_10_CFU/g) was obtained for experiments no. 8 and 10 with a higher dose of CaO_2_ (7.0 and 8.4 wt %, respectively). The number *E. coli* decreased to the acceptable level in experiment no. 12, where the contact time of poultry manure with CaO_2_ was 108 h and process temperature reached 38.8 °C. Comparable result of reduction of *E. coli* was observed in experiment no. 14 (3.28 log_10_CFU/g) with the longest contact time (209 h), where the microbicide concentration in tested sample was 5.0 wt % and temperature was 22 °C. The experiments performed in the centre of the plan, i.e., 15 (C) and 16 (C) for the same values of the input parameters showed the similar number of *E. coli* (4.91 log_10_CFU/g and 4.64 log_10_CFU/g, respectively). Obtained data may suggest that the concentration of CaO_2_ in the poultry manure is crucial for effective decrease the number of bacteria. The aim of the optimization of the microbial inactivation of poultry manure was to eliminate bacteria *E. coli* to maximal value of 3.0 log_10_CFU/g. [19].

The obtained results confirmed the findings of our previous study [50], which had shown that the application of CaO_2_ for the hygienizing process enabled effective reduction of Enterobacteriaceae (coliform bacteria) in poultry manure.

The effect of CaO_2_ used in the poultry industry showed stabilization of microflora and proved that CaO_2_ amendment has no negative impact on the physicochemical parameters of poultry litter [51]. The antimicrobial properties of CaO_2_ compared to Ca(OH)_2_ with wheat seeds was investigated by Sladdin and Lynch [52]. The obtained results suggest that CaO_2_ had potential as a plant protection compound. It improved emergence of wheat in waterlogged soil and did not seem to be toxic. Calcium peroxide was also used as a promising material for hydrogel formation, which showed antibacterial activity by inhibiting the growth of *E. coli* and *Staphylococcus aureus* [53].

However, the literature offers no research, which shows optimizing the parameters of the microbial inactivation of livestock waste using CaO_2_.

The results of the ANOVA test of the inactivation of *E. coli* model, after excluding non-significant linear-linear interaction effects are presented in Table 3. 

The calculated values of the coefficient R^2^ and the adjusted coefficient R_adj._^2^ were 89.0% and 81.7%, respectively. It was proven that the determined regression plan demonstrated good fit of the model to the experimental data. The obtained data suggest that using CCD/RSM method to inactivation pathogens in livestock waste was in agreement with other results. In the case of the real wastewater originating from the textile industry, R^2^ and R_adj._^2^ were 88.0% and 80.0%, respectively [54]. The optimization of application of potassium ferrate (VI) in the treatment of tannery wastewater have revealed R^2^ and R_adj._^2^ values of 77.0% and 59.0% [55]. Furthermore, a Box–Behnken experimental design with RSM was used to optimize condition for microbial reduction on fresh-cut celery [56]. The calculated value of R^2^ for bacteria *E. coli* O157:H7 was 98.0% and for *Salmonella typhimurium* 96.0%, whereas the reduction of pathogenic bacteria was more by 5 log_10_CFU/g. High coefficients R^2^ and R_adj._^2^ i.e., 98.3% and 99.6%, respectively were determined also using RSM experimental design analysis for dyes biodegradation by bacteria *E. coli* [57].

The conducted statistical analysis (Table 3) presented three (except constant value) significant parameters (*p* < 0.05), i.e., CaO_2_ (L), temperature (L) and the contact time (L). Other parameters were statistically insignificant (*p* > 0.05), including CaO_2_ (Q), temperature (Q) and time (Q). Moreover, the results of the analysis showed that the value of mean square error was 0.3877.

**Figure 2:**
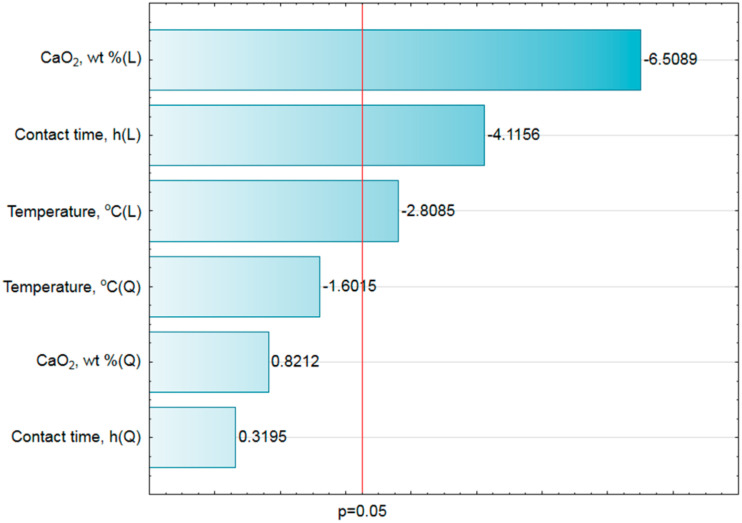
Bar-chart of the absolute value of standardized assessment of the effects (*E. coli*, log_10_CFU/g, 3 value, 1 block, 16 experiments, MS = 0.3877). L—linear effect and Q—quadratic effect.

The verification of the quality of fit of the experimental data to the developed model was presented in graphic form with a Pareto bar chart, which showed the most important factors (Figure 2). 

The lengths of the horizontal bars represent the estimators values of standardized effects from the highest to the lowest while the vertical line shows absolute value of the standardized effect (*p* = 0.05).

The results of the model adequacy verification using an ANOVA test after excluding non-significant linear–linear interaction effects using Statistica 10 is shown in Table 4.

**Figure 3:**
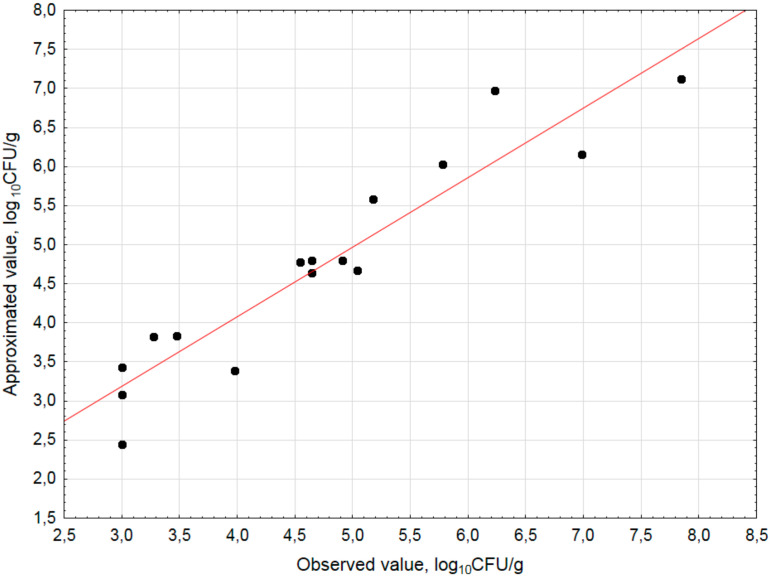
The correlation between the estimated and observed values (*E. coli*, log_10_CFU/g, 3 value, 1 block, 16 experiments, MS = 0.3877).

The data presented in Figure 3 indicated the linear relationship between the observed and approximated values of the number of *E. coli*. 

The data points were close to the red line and shown good adjustment of the experimental values to the predicted values, which suggest the adequacy of the created model.

**Figure 4:**
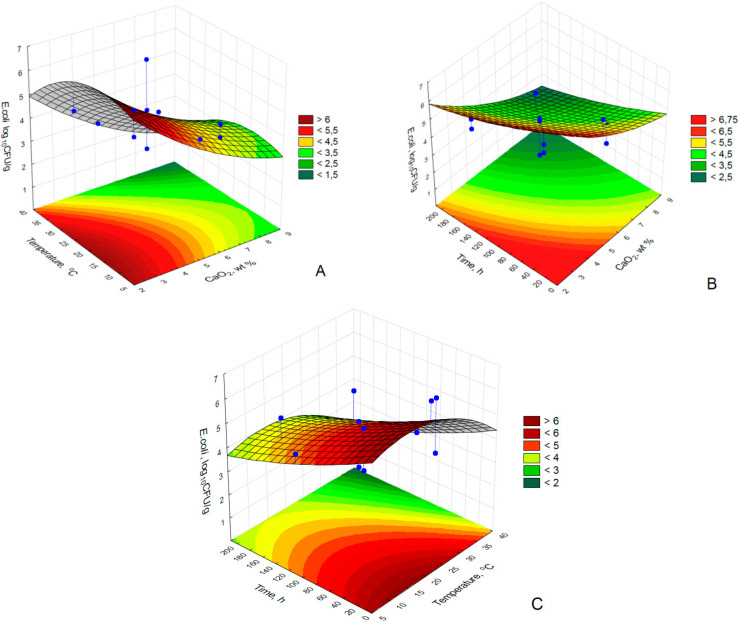
The interactions between: (**A**) temperature (°C) and CaO_2_ concentration (wt %), (**B**) contact time (h) and CaO_2_ concentration (wt %) and (**C**) contact time (h) and temperature (°C).

The graphical illustrations of the regression model as a three dimensional response contour plot were illustrated in Figure 4.

The contour plot shapes describing the influence of independent parameters (CaO_2_ concentration, temperature and contact time) on the number of *E. coli* in the poultry manure. Individual plots show whether the estimated value of one dependent variable interact perfectly with variables of two independent parameter and one constant.

The performed analysis showed that at a constant temperature (x_2_ = 22 °C) the accepted threshold of *E. coli* (3 log_10_CFU/g) was obtained at CaO_2_ > 7 wt % and contact time > 160 h (Figure 4A).

The interaction between the temperature and the microbiocide concentration at constant time (x_3_ = 108 h) was illustrated in Figure 4B. The highest reduction of bacteria took place at the concentration of CaO_2_ > 7 wt % and temperature > 25 °C.

An addition of CaO_2_ to a raw poultry manure at 5 wt %., enabled a reduction of the number of *E. coli* to the 3 log_10_CFU/g after 120 h of the contact time and temperature > 30 °C (Figure 4C).

For a decrease number of *E. coli* in livestock waste to acceptable level of 3 log_10_CFU/g with calcium peroxide as a green oxidizer, the optimal values for each factor were found as follows: 0.08 g of CaO_2_ per 1 gram of poultry manure, temperature 22 °C and contact time 108 h.

The calculated coefficients of the approximating polynomial model for the experimental data is shown in Table 5.

The achieved model for inactivation of *E. coli* bacteria was described as the following Equation (8):

*E. coli* (log_10_CFU/g) = 9.50823 − 0.96835 x_1_ + 0.41999 x_1_^2^ + 0.09683 x_2_ − 0.00327 x_2_^2^ − 0.01547 x_3_ + 0.00002 x_3_^2^ (8)

where x_1_ is CaO_2_ concentration; x_2_ is temperature and x_3_ is contact time.

The statistical analysis indicated that the most important parameter was concentration of CaO_2_ but the important role of temperature and contact time in inactivation of bacteria *E. coli* were also noticed.

Literature review showed that *E. coli* contained in soils amended with animal manure can survive from several weeks to several months. Wang et al. [58] proved that at a temperature 37 °C *E. coli* was destroyed after 42–49 days, at a temperature 22 °C after 49–56 days and the survival time of it at low temperature 5 °C was in the range of 63–70 days. The reduction of *E. coli* in peaty soil with addition of cattle slurry at 4 °C and 20 °C was observed after 30 and 26.8 weeks, respectively [59].

Recent research showed that the most harmful of *E. coli* strains is O157:H7, which causes human and animal diseases. Even 10 cells of this bacteria may be sufficient to cause a serious infection [60]. *E. coli* O157:H7 is dangerous because of its high pathogenicity and acid-resistance properties (pH 2.5), which allows passage through the stomach [10,61]. It was reported that survival time of *E. coli* O157:H7 at storing temperature of 5–30 °C was 10–100 days [62]. The effect of temperature on survival time of *E. coli* O157:H7 in livestock manure compost was reported also by Jung et al. [63]. The results indicated that pathogen was be able to persist 1 day at 50 °C, 120 days at 35 °C and 140 days at 25 °C. According to Jiang et al. [64] in manure-amended soils bacteria *E. coli* O157:H7 can survive even in very dry conditions where moisture of soil is less than 1%. For that reason, the effective reduction of *E. coli* presence in poultry manure requires new sophisticated methods (e.g., using green oxidizing agents, such as calcium peroxide), which can offer additional benefits for plant growth and environment.

### 3.3. Effect of Inactivation of Poultry Manure Treated with CaO_2_ on a Grass Seed Mixture

The obtained results of phytotest indicated different effect of tested soils on the germination and growth of a grass seed mixture (see Table 6).

The average weight of the plants grown on the soil S_1_, before and after drying at 70 °C, was 0.447 g and 0.095 g respectively, and increased with the addition of poultry manure to 0.966 g and 0.187 g for S_1_ + 1% PM and 1.194 g and 0.231g for S_1_ + 2% PM. A visible growth in their mass was also observed for the soil S_2_. The determined mass for the control sample amounted from 0.879 to 2.221 g (S_2_ + 1% PM) and 2.229 g (S_2_ + 2% PM) before drying and from 0.201 to 0.370 g (S_2_ + 1% PM) and 0.316 g (S_2_ + 2% PM) when dried.

The conducted test of the increase of the mixture of grass on the soils enriched with poultry manure combined with CaO_2_ had no effect on the discoloration of leaves or the change in their color. 

**Figure 5:**
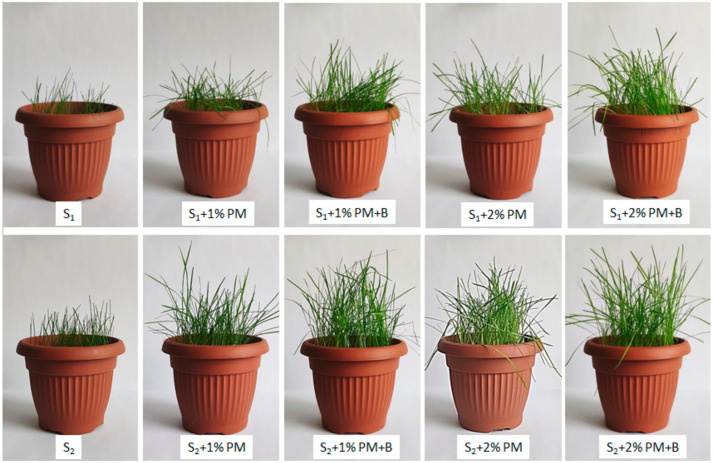
Pot samples with the grass seed mixture after 21 days of plant growth.

The necrosis of the plants was not observed nor were any other changes indicating a negative impact of this substance on the plant growth (see Figure 5).

Moreover, the analysis of the impact of the CaO_2_ addition showed the stimulating effect on the plant growth in comparison to the plants grown on grounds amended with PM. For the S_1_ the mass of the plants growing on the soils enriched with the microbiocidal agent before drying amounted to 1.790 g (S_1_ + 1% PM + B) and 1.837 g (S_1_ + 2% PM + B), whereas the plant mass for the soil S_2_ + 1% PM + B and S_2_ + 2% PM + B reached 2.221 g and 2.811 g respectively.

**Figure 6:**
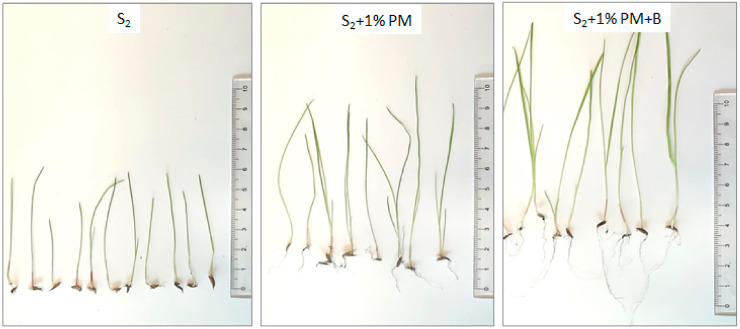
Impact of poultry manure treated with calcium peroxide on roots and shoots length of the grass seed mixture.

In all the cases, the plant growth rates obtained on the soils amended with CaO_2_ additive were characterized with higher quality of the harvest in terms of both the biomass weight and the length of the shoot and the root (Figure 6).

The measured length of the roots of the plants grown on the tested grounds fell within the rage from 0.1 to 0.4 cm (for S_1_) and 0.1 to 0.5 cm (for S_2_), with the length of the shoots respectively at: 1.2–7.8 cm (for S_1_) and 3.0–9.3 cm (for S_2_). The measured length of the roots of the plants grown on the tested grounds with addition of poultry manure ranged from 0.1 to 1.3 cm (for S_1_ + PM) and 0.8 to 4.2 cm (for S_2_ + PM) and was lower compared to the CaO_2_-amended soils, i.e., 0.5–5.2 cm (for S_1_ + PM + B) and 1.4–7.1 cm (for S_2_ + PM + B). The measured length of the shoots on poultry-manure deactivated soils amounted to 3.6–11.9 cm (for S_1_ + PM) and 6.9–16.4 cm (for S_2_ + PM). The CaO_2_-amended shoot length was higher ranging from 5.0 to 13.5 cm (for S_1_ + PM + B) and 6.9 to 16.4 cm (for S_2_ + PM + B).

The determined root growth coefficient (GFR) for soils treatment with CaO_2_ was at: 85.44% (for S_1_ + 1% PM + B), 94.68% (for S_1_ + 2% PM + B), 95.63% (for S_2_ + 1% PM + B) and 93.08% (for S_2_ + 2% PM + B). The determined shoot growth coefficient—GFS amounted to 43.17% (for S_1_ + 1% PM + B), 51.65% (for S_1_ + 2% PM + B), 47.33% (for S_2_ + 1% PM + B) and 49.89% (for S_2_ + 2% PM + B). The coefficients: GFR and GFS for the soils without amendments were lower, reaching the following values: 77.94% and 36.27% (for S_1_ + 1% PM); 79.17% and 37.47% (for S_1_ + 2% PM); 89.51% and 42.67% (for S_2_ + 1% PM) and 92.16% and 45.14% (for S_2_ + 2% PM).

In addition, it was noted that in S_2_ soil, the increased amount of PM additive at 2 wt. % did not affect plant growth as significantly as in S_1_ soil. The content of heavy metals in the soils also had an impact on the plant growth and development. The concentration of toxic heavy metals such as Zn, Pb or Cd (Table 1) may result in less plant growth in soil S_1_ compared to soil S_2_.

The reason for better growth of grass plants can be linked to the increasing availability of oxygen generated from CaO_2_. It can provide oxygen through the soil supporting quick growth of root systems and decontaminates the seeds. According to the stoichiometric equation (2) the maximal amount of H_2_O_2_ formed through CaO_2_ is 0.47 g H_2_O_2_/g CaO_2_ [31].

The result of the germination with 20 g/kg of CaO_2_ directly on vegetable seeds was presented by Domaradzki et al. [30]. On the basis of the tests, it was found that the germination of some kinds of seeds had improved significantly.

Most investigations showed that seeds pelleting with calcium peroxide (CaO_2_) promoted germination of rice and improved the plant growth [65,66,67]. It has been reported that seed rice (*Oryza sativa* L.) coating with CaO_2_ resulted in better germination, higher yield of rice plants (85%) and reduced mean emergence time of dry direct seeded rice [68].

The decreasing pH in organic fertilizers may affect the pH of the soils and the composition of bioavailable forms of macro and micro nutrients and heavy metals. According to our previous study [50] the pH value of fresh poultry manure (pH = 6) was increasing with amendment of calcium peroxide from pH = 7 (for 3.5 wt % CaO_2_) to pH = 10.5 (for 10.5 wt % CaO_2_). Our research leads to the conclusion that by being affected by CaO_2_, the organic fertilizers and the soil are subject to slight alkalization. Under these conditions, metal ions precipitate in the form of insoluble hydroxides (e.g., Pb(OH)_2_), which cannot be absorbed by plant roots. As a consequence, the amount of free metal ions in the soil is also reduced. This may be the additional reason for the better growth of the plants used in tests.

Using CaO_2_ as an amendment to livestock waste allows one to decrease pathogens to a safe level and stimulates the plant growth. Furthermore, it is recognized as ecological friendly and green oxidizing compound because of its luck of odor, easy biodegradability in soil and due to the absence of harmful decomposition products.

## 4. Conclusions

Inactivation of *E. coli* in raw poultry manure using traditional calcium compounds (CaO or Ca(OH)_2_) requires long storage time, temperature higher than 50 °C and pH value 12–13. Applying CaO_2_ as an amended to livestock wastes allows to decrease the values of temperature and time and neutralize the hygienization process.

The effective reduction of *E. coli* to an acceptable level, i.e., below 1000 CFU/g was obtained for CaO_2_ in concentration 5 wt % in PM, with temperature more than 38 °C and contact time 108 h or CaO_2_ 8 wt %, at a temperature 22 °C and after 108 h. Antimicrobial effect of CaO_2_ is connected with releasing active oxygen without any harmful substances, which makes it the ecologically friendly compound for environment.

Applying of CaO_2_ as an amendment to poultry manure has a positive effect on germination and growth grass seed mixture, improved the properties of the soils and groundwater and may be used for soil reclamation of industry-degraded areas.

## Figures and Tables

**Table 1:** Chemical characterization of soils and poultry manure used in this study.

Parameter	Unit	PM	S_1_	S_2_
Moisture content	%	62.5	0.6	0.6
Ash content	%	12.9	95.4	97.5
TOC	g/kg dm	418.5	nd	nd
S		6.2	0.3	0.3
N		56.7	nd	nd
P		20.1	0.3	0.2
K		23.6	7.2	4.8
Ca		24.0	1.6	0.6
Mg		8.5	0.7	0.2
Na		5.3	0.8	0.1
Si		1.8	413.8	441.8
Al		0.5	15.5	9.5
Fe		0.8	11.0	3.7
Ba	mg/kg dm	370.0	308.8	201.2
Cd		bdl	35.2	12.1
Co		bdl	3.0	5.0
Cr		bdl	12.1	13.1
Cu		68.0	42.3	17.1
Mn		383.0	434.5	133.8
Ni		20.0	10.1	7.0
Pb		bdl	942.5	387.2
Rb		13.0	94.6	30.2
Sr		34.0	3.0	4.0
Zn		428.0	6920.1	746.3
*E. coli*	log_10_CFU/g	8.3	nd	nd

nd—not determined; bdl—below detection limit.

**Table 2:** Empirical conditions and results for the CCD/RSM analysis.

Run	Experimental Conditions			Experimental Results
	CaO_2_ (wt %)	Temperature (°C)	Contact Time (h)	*E. coli* (log_10_ CFU/g)
1	3.0	12.0	48	6.2304
2	3.0	12.0	168	5.1761
3	3.0	32.0	48	5.7782
4	3.0	32.0	168	4.6435
5	7.0	12.0	48	4.5441
6	7.0	12.0	168	3.9777
7	7.0	32.0	48	3.4771
8	7.0	32.0	168	3.0000
9	1.6	22.0	108	7.8451
10	8.4	22.0	108	3.0000
11	5.0	5.2	108	5.0414
12	5.0	38.8	108	3.0000
13	5.0	22.0	7	6.9868
14	5.0	22.0	209	3.2788
15 (C) *	5.0	22.0	108	4.9085
16 (C) *	5.0	22.0	108	4.6435

* experiments in the center of the plan.

**Table 3:** Analysis of the inactivation *E. coli* model with CCD/RSM using ANOVA model coefficients.

Parameter	The Evaluation of the Effects, *E. Coli* log_10_CFU/g; R^2^ = 0.89008, R^2^_adj_ = 0.8168, 3 Parameters, 1 Block, 16 Experiments, MS = 0.3877								
	Effect	Standard Error	*p*-Value *	−95% Confidence Interval	+95% Confidence Interval	Factor	Standard Error of Factor	Lower Confidence Interval	Upper Confidence Interval
Constant value	4.80110	0.439007	0.000002	3.80799	5.79420	4.80110	0.439007	3.80799	5.794202
CaO_2_, wt % (L)	−2.19344	0.336989	0.000110	−2.95576	−1.43112	−1.09672	0.168495	−1.47788	−0.715559
CaO_2_, wt % (Q)	0.33599	0.409156	0.432751	−0.58958	1.26157	0.16800	0.204578	−0.29479	0.630785
Temperature, °C (L)	−0.94644	0.336989	0.020426	−1.70877	−0.18412	−0.47322	0.168495	−0.85438	−0.092060
Temperature, °C (Q)	−0.65526	0.409156	0.143731	−1.58084	0.27031	−0.32763	0.204578	−0.79042	0.135156
Contact time, h (L)	−1.38691	0.336989	0.002615	−2.14924	−0.62459	−0.69346	0.168495	−1.07462	−0.312295
Contact time, h (Q)	0.13072	0.409156	0.756645	−0.79485	1.05630	0.06536	0.204578	−0.39743	0.528149

L—linear effect; Q—quadratic effect; * statistically significant (*p* < 0.05) and statistically insignificant (*p* > 0.05).

**Table 4:** Analysis of variance (ANOVA) of inactivation *E. coli* model with CCD/RSM.

Parameter	Assessment of the Effects, *E. Coli* log10CFU/g; R^2^ = 0.89008, R^2^_adj_ = 0.8168, 3 Parameters, 1 Block, 16 Experiments, MS = 0.3877				
	SS	DF	MS	F	*p*-Value *
CaO_2_, wt % (L)	16.4264	1	16.4264	42.3662	0.0001
CaO_2_, wt % (Q)	0.2615	1	0.2615	0.6744	0.4328
Temperature, °C (L)	3.0583	1	3.0583	7.8878	0.0204
Temperature, °C (Q)	0.9944	1	0.9944	2.5648	0.1437
Contact time, h (L)	6.5673	1	6.5673	16.9382	0.0026
Contact time, h (Q)	0.0396	1	0.0396	0.1021	0.7566
Error	3.4895	9	0.3877	-	-

L—linear effect, Q—quadratic effect, SS—predicted residual error of sum of squares, DF—degree of freedom, MS—mean square error, F—statistics and * statistically significant (*p* < 0.05).

**Table 5:** Regression coefficients of the inactivation *E. coli* model.

Predictor	Regression Coefficient	Standard Error	t-Value, df * = 9	*p*-Value **	−95% Confidence Interval	+95% Confidence Interval
Intercept	9.508227	2.024962	4.69551	0.001127	4.92744	14.08901
CaO_2_ (L)	−0.968354	0.518337	−1.86819	0.094571	−2.14091	0.20421
CaO_2_ (Q)	0.041999	0.051145	0.82119	0.432751	−0.07370	0.15770
Temperature (L)	0.096836	0.091578	1.05741	0.317877	−0.11033	0.30400
Temperature (Q)	−0.003276	0.002046	−1.60150	0.143731	−0.00790	0.00135
Time (L)	−0.015479	0.012592	−1.22931	0.250124	−0.04396	0.01301
Time (Q)	0.000018	0.000057	0.31949	0.756645	−0.00011	0.00015

* *df*—degree of freedom; ** statistically significant (*p* < 0.05).

**Table 6:** The effect of PM treated with CaO_2_ on the plant growth and biomass weight of a grass seed mixture.

Soil Sample	Average Lenght of Root * (cm)	Average Lenght of Shoot * (cm)	Weigh of Fresh Biomass ** (g)	Weigh of Dried Biomass ** (g)	GFR (%)	GFS (%)
S1	0.15 ± 0.08	4.99 ± 1.19	0.447 ± 0.050	0.095 ± 0.010	-	-
S1 + 1% PM	0.68 ± 0.30	7.83 ± 1.64	0.966 ± 0.070	0.187 ± 0.030	77.94	36.27
S1 + 1% PM + B	1.03 ± 0.32	8.78 ± 1.72	1.790 ± 0.080	0.292 ± 0.020	85.44	43.17
S1 + 2% PM	0.72 ± 0.27	7.98 ± 1.45	1.194 ± 0.060	0.231 ± 0.030	79.37	37.47
S1 + 2% PM + B	2.82 ± 1.05	10.32 ± 1.73	1.837 ± 0.070	0.346 ± 0.030	94.68	51.65
S2	0.20 ± 0.12	5.93 ± 1.31	0.879 ± 0.040	0.201 ± 0.020	-	-
S2 + 1%PM	1.91 ± 0.66	10.34 ± 2.09	2.221 ± 0.050	0.370 ± 0.030	89.51	42.67
S2 + 1%PM+B	4.58 ± 1.44	11.26 ± 1.66	2.869 ± 0.070	0.445 ± 0.040	95.63	47.33
S2 + 2%PM	2.55 ± 0.78	10.81 ± 1.47	2.229 ± 0.060	0.316 ± 0.020	92.16	45.14
S2 + 2%PM+B	2.89 ± 0.75	11.84 ± 1.44	2.811 ± 0.090	0.415 ± 0.050	93.08	49.89

* average ± standard deviation (*n* = 35), ** average ± standard deviation (*n* = 3).

## Data Availability

Data sharing not applicable.
